# Fatal Invasive Aspergillosis and Coronavirus Disease in an Immunocompetent Patient

**DOI:** 10.3201/eid2607.201603

**Published:** 2020-07

**Authors:** Marion Blaize, Julien Mayaux, Cécile Nabet, Alexandre Lampros, Anne-Geneviève Marcelin, Marc Thellier, Renaud Piarroux, Alexandre Demoule, Arnaud Fekkar

**Affiliations:** Assistance Publique–Hôpitaux de Paris, Groupe Hospitalier La Pitié-Salpêtrière, Paris, France (M. Blaize, J. Mayaux, C. Nabet, A. Lampros, A.-G. Marcelin, M. Thellier, R. Piarroux, A. Demoule, A. Fekkar);; Sorbonne Université, Paris (C. Nabet, A.G. Marcelin, M. Thellier, R. Piarroux, A. Fekkar)

**Keywords:** Aspergillus, SARS-CoV-2, aspergillosis, intensive care unit, mechanical ventilation, fungal superinfection, severe acute respiratory syndrome coronavirus 2, coronavirus disease, COVID-19, SARS, zoonoses, viruses, coronavirus, fungi

## Abstract

Invasive pulmonary aspergillosis is a complication in critically ill patients with acute respiratory distress syndrome, especially those with severe influenza pneumonia. We report a fatal case of invasive pulmonary aspergillosis in an immunocompetent patient in France who had severe coronavirus disease–associated pneumonia.

Patients infected with severe acute respiratory syndrome coronavirus 2 (SARS-CoV-2) experience major lung damage due to viral replication and the ensuing cytokine storm and complex inflammatory processes (*1*). The severe damage to lung tissue can lead to secondary infections within a median of 17 days after onset of coronavirus disease (COVID-19) (*2*). Most immunocompetent patients who develop severe forms of COVID-19 have >1 underlying condition, such as chronic obstructive pulmonary disease, hypertension, diabetes, or chronic kidney disease (*3*), but none of these predisposing factors generally are associated with an increased risk for developing fungal infections.

Invasive aspergillosis is a well-described complication of severe influenza pneumonia (*4*,*5*), but many intensivists seem to overlook this superinfection (*6*). A recent study reported a 19% incidence among 432 patients admitted to an intensive care unit (ICU) for influenza-related acute respiratory failure (*4*). Moreover, in a small autopsy series of patients who died in 2003 from SARS, 10% (2/20) had an invasive infection suggestive of aspergillosis (*7*). As the medical community confronts the ongoing COVID-19 pandemic, determining whether patients infected with SARS-CoV-2 develop fungal complications, especially invasive aspergillosis, is crucial.

We report the case of a 74-year-old immunocompetent man with severe COVID-19–associated pneumonia who rapidly developed invasive pulmonary aspergillosis. The patient had several underlying chronic diseases but no pulmonary disease. Among his existing conditions were asymptomatic and untreated myelodysplastic syndrome, diagnosed on the basis of hypereosinophilia, with CD8+ T-cell lymphocytosis, a normal karyotype and low-risk International Prognostic Scoring System score; Hashimoto’s thyroiditis; hypertension; and benign prostatic hypertrophy. 

On the morning of March 23, day 1, the patient fell out of bed, was unable to get up on his own, and called emergency services. The patient described a 1-week history of fever and cough followed by onset of dyspnea that day. Clinical examination showed signs of acute respiratory failure, tachypnea, and hypoxemia. The patient was intubated, transported to the hospital, and transferred to the ICU, where he required mechanical ventilation and vasopressor support. The same day, we took a protected distal aspiration before intravenous administration of cefotaxime. SARS-CoV-2 viral RNA was detected in this sample by using 2 distinct reverse transcription PCRs. The sample revealed 10^4^/mL of *Haemophilus influenzae*, which was not found in a second protected distal aspiration performed on day 2.

The patient’s condition worsened, and he had a PaO_2_/FIO_2_ ratio <140 mm Hg with FIO_2_ of 100%. We performed a tracheal aspirate on day 4 and sent samples to the mycology laboratory. PCR for *Aspergillus fumigatus* was positive (430 copies/mL; 2.6 log), but galactomannan in the aspirate assay was negative, and culture on Sabouraud agar remained sterile. Results of testing of a second tracheal aspiration performed on day 9 yielded branched septate hyphae suggestive of *Aspergillus* sp. under microscopic examination after silver staining (Figure). *A. fumigatus* PCR was once again positive with a higher number of copies (3,600 copies/mL; 3.55 log); the galactomannan assay was not available. The patient died that day of severe respiratory failure. Culture on Sabouraud agar later grew a mold identified as *A. fumigatus* by mass spectrometry. Of note, SARS-CoV-2 RNA still was detectable in the tracheal aspirations. Results of serum analyses, including galactomannan index determination, β-D-glucan assay, and *A. fumigatus* PCR, were negative in serum samples collected on day 4 and day 8.

**Figure:**
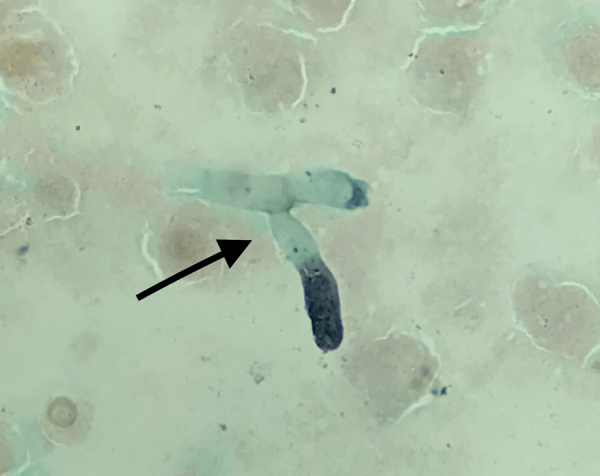
Microscopic image of silver-stained tracheal aspirate from an immunocompetent patient critically ill with coronavirus disease, France. Filaments suggestive of *Aspergillus* sp. are shown (black arrow). Original magnification ×1,000.

This case fulfills the criteria of putative invasive aspergillosis according to the classification defined by Blot et al. for the diagnosis of invasive pulmonary aspergillosis in critically ill patients (*8*). Because the patient’s illness took a rapid and fatal course, we did not have time to initiate appropriate antifungal treatment. As previously reported, respiratory samples led to a diagnosis of aspergillosis, but blood samples lacked sensitivity (*4*). Moreover, *Aspergillus* PCR contributed to the diagnosis, but the galactomannan was negative, consistent with what we previously reported about the lower sensitivity of the galactomannan compared with PCR and culture in respiratory samples (*9*).

A patient with a fatal SARS-CoV-2 infection recently was reported to have *A. flavus* on tracheal aspirates culture, but other evidence was missing, and diagnosis of aspergillosis was not substantiated (*10*). Additional assessments are urgently needed to thoroughly investigate cases and promptly establish and define the cumulative incidence of invasive aspergillosis in ICU patients with severe COVID-19.

In conclusion, we report a case of invasive pulmonary aspergillosis in an immunocompetent patient during severe COVID-19–associated pneumonia. As the COVID-19 outbreak continues to spread worldwide, other reports are needed to assess the occurrence and frequency of fungal complications during severe SARS-CoV-2 infections.
